# AN EVALUATION OF THE VA RURAL INTERDISCIPLINARY TEAM TRAINING PROGRAM: OUTCOMES AND IMPLICATIONS

**DOI:** 10.1093/geroni/igz038.2815

**Published:** 2019-11-08

**Authors:** Judith L Howe, Josea Kramer, Nancy Weintraub, Eve Gottesman, Regina Richter, Andrew Bean

**Affiliations:** 1 James J Peters VA Medical Center, GRECC, Bronx, New York, United States; 2 Greater Los Angeles Health Care System GRECC, North Hills, California, United States; 3 James J. Peters GRECC, Bronx, New York, United States; 4 David Geffen School of Medicine, UCLA, North Hills, California, United States

## Abstract

The rural interdisciplinary team training (RITT) program has provided in-person training for almost 2000 VA providers and staff at 125 rural clinics since 2011. The multi-modal workshop, accredited for 6.5 hours for a number of disciplines, focuses on the recognition of common issues facing older Veterans, red flags prompting further assessment, how to administer screening instruments and team-based approaches for improving patient outcomes. Participants develop an improvement action plan improvement project based on common challenges faced by clinic patients. A program evaluation found an increase in geriatrics knowledge and a modest improvement in teamwork after the education program. It also found that participants self-identified an enhanced ability on average to use red flags after the 6.5 hour training in areas such as polypharmacy, falls and caregiver stress. The action plans were often not implemented at follow-up. The evaluation results will be discussed, as well as challenges, limitations, and implications.

